# Exploring Consensus on Preventive Measures and Identification of Patients at Risk of Age-Related Macular Degeneration Using the Delphi Process

**DOI:** 10.3390/jcm10225432

**Published:** 2021-11-20

**Authors:** Alfredo García-Layana, Gerhard Garhöfer, Tariq M. Aslam, Rufino Silva, Cécile Delcourt, Caroline C. W. Klaver, Johanna M. Seddon, Angelo M. Minnella

**Affiliations:** 1 Clínica Universidad de Navarra, IdiSNA (Instituto de Investigación Sanitaria de Navarra), 31009 Pamplona, Spain; aglayana@unav.es; 2 Department of Clinical Pharmacology, Medical University of Vienna, 1090 Wien, Austria; 3 School of Pharmacy and Optometry, Faculty of Biology, Medicine and Health, University of Manchester, Manchester M13 9PL, UK; tariq.aslam@manchester.ac.uk; 4 Manchester Royal Eye Hospital, Manchester University NHS Foundation Trust, Manchester M13 9WL, UK; 5 Faculty of Medicine, Institute for Clinical and Biomedical Research (ICBR-FMUC), University of Coimbra, 3000-548 Coimbra, Portugal; rufino.silva@oftalmologia.co.pt; 6 Ophthalmology Department, Centro Hospitalar e Universitário de Coimbra (CHUC), 3004-561 Coimbra, Portugal; 7 AIBILI-Association for Innovation and Biomedical Research on Light and Image (AIBILI), 3000-548 Coimbra, Portugal; 8 University Bordeaux, Inserm, Bordeaux Population Health Research Center, Team LEHA, UMR 1219, F-33000 Bordeaux, France; cecile.delcourt@u-bordeaux.fr; 9 Department Ophthalmology, Erasmus Medical Center, 3000 CA Rotterdam, The Netherlands; c.c.w.klaver@erasmusmc.nl; 10 Department Epidemiology, Erasmus Medical Center, 3000 CA Rotterdam, The Netherlands; 11 Department Ophthalmology, Radboud University Medical Center, 6525 GA Nijmegen, The Netherlands; 12 Institute for Molecular and Clinical Ophthalmology, CH-4031 Basel, Switzerland; 13 Department of Ophthalmology and Visual Sciences, University of Massachusetts Medical School, Worcester, MA 01655, USA; johanna.seddon@umassmed.edu; 14 UOC Oculistica, Università Cattolica del Sacro Cuore, Fondazione Policlinico Universitario A. Gemelli, IRCCS, 00168 Rome, Italy; angelomaria.minnella@unicatt.it

**Keywords:** age-related macular degeneration, prevention, identification, risk, Delphi, STARS^®^, food supplement

## Abstract

Background: Early identification of AMD can lead to prompt and more effective treatment, better outcomes, and better final visual acuity; several risk scores have been devised to determine the individual level of risk for developing AMD. Herein, the Delphi method was used to provide recommendations for daily practice regarding preventive measures and follow-up required for subjects at low, moderate, and high risk of AMD evaluated with the Simplified Test AMD Risk-assessment Scale (STARS^®^) questionnaire. Methods: A steering committee of three experts drafted and refined 25 statements on the approach to be recommended in different clinical situations [general recommendations (*n* = 2), use of evaluation tools (*n* = 4), general lifestyle advice (*n* = 3), and AREDS-based nutritional supplementation (*n* = 5)] with the help of a group of international experts, all co-authors of this paper. Thirty retinal specialists from Europe and the US were chosen based on relevant publications, clinical expertise, and experience in AMD, who then provided their level of agreement with the statements. Statements for which consensus was not reached were modified and voted upon again. Results: In the first round of voting, consensus was reached for 24 statements. After modification, consensus was then reached for the remaining statement. Conclusion: An interprofessional guideline to support preventive measures in patients at risk of AMD based on STARS^®^ scoring has been developed to aid clinicians in daily practice, which will help to optimize preventive care of patients at risk of AMD.

## 1. Introduction

Age-related macular degeneration (AMD) is a primary reason for blindness in Western countries [1,2,3]. The number of individuals with AMD is believed to rise globally by around 40% from 2020 to 2040, highlighting the need for effective preventive measures and therapies [2]. The two late phenotypes of AMD, neovascular and atrophic AMD, are usually preceded by drusen and pigmentary abnormalities.

Considerable progress has been made in the treatment of AMD, and in this regard, intravitreal injections of antiangiogenic agents have greatly changed the management of neovascular AMD [3]. While these agents can provide stabilization or even rapid improvement of visual acuity in the majority of patients at the start of treatment, the long-term results regarding visual acuity and quality of life remain uncertain [4,5,6]. Moreover, there is still no therapy for geographic atrophy, which represents a considerable proportion of cases of late AMD. This confirms that despite therapeutical progress, late-stage AMD remains a major cause of visual loss. Early identification of patients at risk of AMD is thus of significant clinical relevance, as it can lead to more effective treatment, better outcomes, and better visual acuity [7].

Epidemiological studies have unveiled the existence of several risk factors for AMD, which include smoking, diet, family history of AMD, and cardiovascular disease, as well as both clinical and ocular risk factors. Several of these risk factors have shown strong associations with AMD (especially smoking, ethnicity, and family history of AMD), although for others such as gender and iris color the results have been less consistent [3,8,9]. In addition, more than 50 genetic polymorphisms have been identified that contribute to the disease [9,10].

Conversely, many endogenous and exogenous micronutrients have been implicated in the protection of the retina against AMD through different mechanisms including antioxidant, anti-inflammatory, and blue light absorption [9,11,12,13,14,15,16,17,18]. Animal models and cell culture studies have suggested that oxidative stress to the retina is an important factor contributing to AMD, and large interventional studies have shown that antioxidant nutrients given as supplements appear to exert a protective effect against progression to advanced forms of AMD [18,19,20,21,22]. Altogether, it is now generally accepted that adequate intake of micronutrients is crucial for a healthy retina and vitamin supplementation needs to be considered in the management of patients with late-stage or progressing AMD [23,24,25,26,27].

Early identification of AMD is of significant clinical relevance, as it can lead to prompt and more effective treatment, better outcomes, and better final visual acuity [7]. In this regard, risk scores have been devised to establish the risk for AMD, thereby allowing for individualized management [28,29,30,31,32,33,34,35]. Even if some of these models have good discrimination for AMD, most include assessment of genetic polymorphisms, which are not used on a routine basis in clinical practice. While clinical, lifestyle, and ocular risk factors have been related to AMD, few risk scores have included dietary considerations [14,29,31,32,33].

Given the need for early identification of individuals at risk and improving disease outcomes, an easy to compile self-administered 13-item questionnaire to evaluate individual risk for AMD in daily practice has been developed (Simplified Test AMD Risk-assessment Scale—STARS^®^) [36]. The scoring system was derived from an initial sample of 12,639 Italian subjects and subsequently validated on 6897 French subjects. The questionnaire showed good discrimination, allowing stratification of subjects according to the risk of AMD (low, moderate, high). The translational relevance of the STARS^®^ score in predicting macular function in early and intermediate AMD has been recently pointed out: STARS^®^ is able to predict central retinal function with a high degree of accuracy, as assessed by full-field electroretinogram, which suggests that both parameters can be combined to assess the clinical risk for loss of visual function even in the early disease stages [37].

Following the development and validation of the score, a process was activated to develop specific recommendations regarding the preventive measures and ophthalmological follow-up to adopt, tailored according to the individual level of risk for AMD that can complement existing guidelines. This is also important considering that validated recommendations can help ophthalmologists to individualize consultations with patients and save time.

Herein, the Delphi method [38] has been used to provide recommendations for the preventive measures to adopt in individuals at low, moderate, and high risk of AMD based on STARS^®^.

## 2. Materials and Methods

### 2.1. Delphi Process

The consensus process was carried out with a three-step Delphi method [39,40]. The Delphi method has been used in healthcare settings as a good means of obtaining consensus [38,41,42,43,44,45]. The method is a consecutive process that uses repeated rounds of voting and is effective in obtaining expert consensus for which there is limited evidence [42].

**Figure 1:**
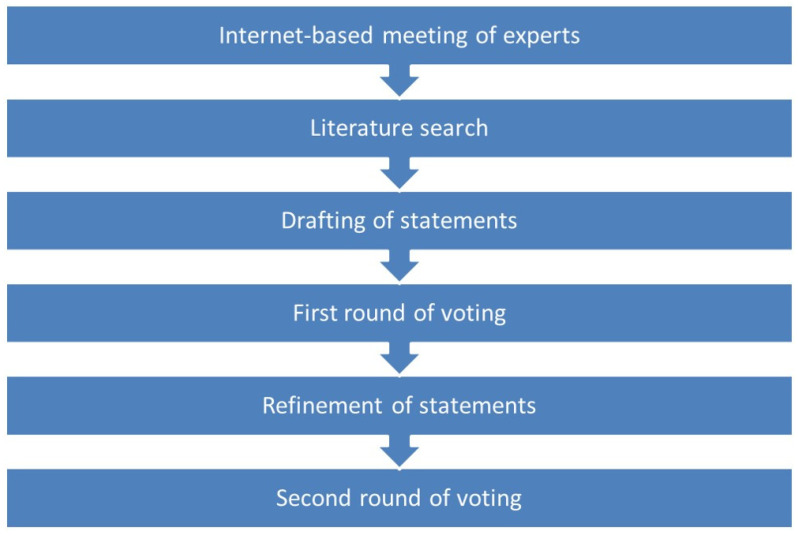
Overview of Delphi process used.

The overall Delphi process adopted is summarized in Figure 1. In March 2019, in the first step, a steering committee composed of three experts drafted a series of statements through a web-based meeting. The scientific committee, a group of international experts and co-authors of this paper, then further refined the statements until final approval of a list of 25 statements, regarding the best approach to recommend in patients with diverse levels of risk for developing AMD based on STARS^®^ score (low, score 0–9; moderate, score 10–19; high, score ≥ 20), age (50–70 years, >70 years) and AREDS categories [46]. STARS^®^ is based on patient data (age, sex, and ethnicity), family history of AMD, medical history, and eye characteristics [36].

For the second step of the Delphi process, retinal specialists from across Europe and the US were chosen based on relevant publications, clinical expertise in the retina, and experience in AMD. Thirty retinal experts were contacted via email and asked to take part in the consensus project, and 24 participants (75%) agreed to participate (France, 2; Spain, 4; UK, 3; Germany, 1; Portugal, 2; Russia, 3; Ukraine, 1; Italy, 2; Poland 2; Turkey, 2; Belgium, 1; USA, 1). The first round of voting took place in July 2019 via a dedicated, password-protected online platform. The steering committee analyzed the results of voting and modified the statements for which agreement was not obtained, based on feedback from the participants. The second round of voting took place in January 2020.

### 2.2. Analysis of Voting and Determination of Agreement

Participants were requested to rate their agreement with the statements proposed on a scale from 1 to 9, where 1 is complete disagreement and 9 is complete agreement. Ratings of 1–3 were classified as disagreement, while ratings of 7–9 were classified as agreement. Ratings of 4–6 were classified as neutral.

Disagreement was assessed as follows. First, the value of inter-percentile range (IPR) was calculated, i.e., the range of responses between the 70th and the 30th percentiles. Next, the value of the inter-percentile range adjusted for symmetry (IPRAS) was calculated, which assesses dispersion for asymmetric distributions; finally, the values of IPR and IPRAS were calculated. Disagreement was considered when IPR > IPRAS [46].

Disagreement inevitably produced an uncertain decision. If, on the other hand, there was no disagreement, the median determined if the agreement was positive, negative, or uncertain. If the median was from 7–9, then a positive is obtained, and the statement was considered relevant for the management of AMD. If the median was from 1–3, then the decision was negative, and the statement was not considered relevant for the management of AMD. A median that was within the 4–6 range produced an uncertain decision.

### 2.3. Ethics Approval

No formal ethics approval was required. All participants agreed to participate, and the data are presented at the group level only. As such, it is not possible to identify an individual participant.

## 3. Results

The Steering Committee, with the help of the scientific committee, all co-authors of this paper, drafted a total of 25 statements in different areas, comprising general recommendations (*n* = 2), use of evaluation tools (*n* = 4), general lifestyle advice (*n* = 3), and AREDS-based nutritional supplementation (*n* = 5) (Table 1). In addition, five statements were proposed for subjects at moderate risk of AMD, and six statements for those at high risk (Table 2). Each Delphi panel expert then voted on his/her level of agreement with each statement using an online platform. Participants had no access to the decisions of the other experts. Consensus, considered when IPR < IPRAS, was reached for 24 of the 25 statements in the first round of voting for statements involving general issues, evaluation tools, lifestyle advice, and AREDS-based nutritional supplementation.

In the first round of voting, consensus (IPR < IPRAS) was reached for all statements, except for statement 12, which had IPR > IPRAS and reached a median of 5 in voting (Table 1, statement 12A). This statement had been initially worded as “An AREDS-based formulation has no significant benefit on the progression of dry AMD or development of geographic atrophy in the long term”. While some experts commented that more evidence is needed, others said that there is sufficient data to recommend the use of an AREDS-based formulation in dry AMD. Based on this, the statement was reworded as “An AREDS-based formulation may have benefit on the progression of dry AMD or development of geographic atrophy in the long term”. After a second round of voting, in which 19 (79%) Delphi panel experts participated, consensus was reached for this reworded statement (12B in Table 1). Eventually, all statements received positive agreement (median ≥ 7), except for statement 16 which was judged of uncertain relevance (median of votes 6.5).

## 4. Discussion

The present Delphi consensus had the main objective of formulating a series of validated recommendations on ophthalmological follow-up and preventive measures to adopt for subjects with a low, moderate, and high risk of developing AMD evaluated with the STARS^®^ questionnaire, which can be useful for ophthalmologists in daily practice. The Delphi process was chosen with the aim of obtaining a consensual response from international panel of experts as there is limited evidence available [43]. While absolute agreement is rarely obtained, the Delphi methodology helps to identify a group consensus [43]. Consensus was reached for 24 of the 25 statements proposed in the first round of voting. The results of voting for each of the three main areas are discussed below.

### 4.1. General Recommendations

Statement #1 addressed the use of intravitreal injections as the first choice in the treatment of patients with wet AMD to stop disease progression as supported by expert recommendations following diagnosis of choroidal neovascularization (CNV) [47], and fully recommended by the European Society of Retina Specialists (EURETINA) [48]. Some of the experts commented that the utility of intravitreal injections is not only to halt the progression of the disease, but also to increase visual acuity and quality of life as much as possible.

Statement #2 addressed the use of nutritional supplements at an early phase of AMD to help reduce the risk of progression in patients at a high risk of AMD. Indeed, there is now general agreement that the positive effects of the AREDS1 and AREDS2 formulations are a result of their antioxidant properties [49], and, based on the AREDS studies, a number of dietary supplements are currently available. Such formulations, in addition to the diet and healthy lifestyle recommendations below, are now considered to be the standard of care to reduce the risk of reaching advanced AMD among those with an elevated risk of progressing to severe visual loss [50].

### 4.2. Use of Evaluation Tools

Early identification of high-risk subjects using a simple tool is a highly desirable goal such that appropriate subjects can be offered ophthalmological follow-up (especially for early diagnosis and treatment of neovascular AMD, as needed) (Statement #3). Moreover, the STARS^®^ questionnaire was considered to be a valid tool to assess the risk of AMD in the general population (statement #4). STARS^®^ is a simple and easy-to-use 13-item questionnaire based on the presence of risk factors auto-administered by patients [36]. The STARS^®^ questionnaire has been validated in two large European observational cohorts and shows good discrimination of risk of AMD into low, moderate, and high-risk categories [36]. As such, STARS^®^ would appear to meet the ideal criteria for a simple screening tool for daily practice.

It was further held that stratification according to the risk of AMD is useful in order to plan lifestyle interventions, give dietary advice, and plan ophthalmological follow-up using STARS^®^ and the AREDS category score (statement #5). Both scoring systems provide simple risk categories and are complementary since the AREDS classification relies only on retinal alterations identified at clinical examination, while the STARS^®^ score relies only on an auto-administered evaluation of risk factors [46]. Several recent studies have indicated that consumption of a Mediterranean diet appears to offer some benefits against developing AMD, possibly through increased intake of micronutrients and antioxidants [14,15,51,52]. In addition, physical activity and weight control are receiving increased attention for their possible role in the prevention of AMD [27]. The last statement on evaluation tools considered that ophthalmologists should use the STARS^®^ and AREDS category score in daily practice to evaluate the risk of AMD and to define the best prevention strategy and follow-up (statement #6). This recommendation is based on the above considerations.

### 4.3. General Lifestyle Advice

Consensus was reached for all the statements on general lifestyle advice, advocating that all subjects who are at risk for AMD should stop smoking, adopt a Mediterranean diet, and carry out regular physical activity (statement #7) as documented in the literature [9,14,15,27,52,53]. In addition, increased intake of vegetables, fruit and fish should be actively encouraged in the aging population, considering that <4% of individuals ≥ 55 years of age had adequate intake of these food groups in an epidemiological study performed in the Netherlands (statement #8) [17,54]. The benefits of dietary omega-3 fatty acid and fish intake in reducing the risk of AMD, for example, appear to be well consolidated as demonstrated by several studies and summarized in a meta-analysis by Chong et al. [55]. This is in contrast to the unexpected, negative results of the AREDS2 study in terms of the benefits of omega-3 fatty acids and AMD [19]. Possible explanations for this, as noted by other authors, include the complex study design. AREDS2 subjects were already taking AREDS1 supplements, which together with the lack of a placebo group for comparison, may not have allowed for the effects of omega-3 fatty acids to be sufficiently evident [18].

Furthermore, smoking is considered to be an important modifiable risk factor for the development and progression of AMD [56,57]. All patients at risk should therefore be actively encouraged to stop smoking. The last statement on general lifestyle advice considered that if patients are unable or unwilling to follow a diet rich in green leafy vegetables, fruits, and fish, such as the Mediterranean diet, nutritional supplements should be recommended in subjects at high risk of AMD (statement #9).

### 4.4. AREDS-Based Supplementation

There were five statements on AREDS-based supplementation. The experts considered that a nutritional formulation reduces the risk of developing advanced AMD in the long-term (statement #10) and that it decreases the overall risk of moderate vision loss in the long term (statement #11). Statement #12A was initially worded as “An AREDS-based formulation has no significant benefit on the progression of dry AMD or development of geographic atrophy in the long term”, for which agreement was not reached. The statement was then modified to “An AREDS-based formulation may have benefit on the progression of dry AMD or development of geographic atrophy in the long term”, and after a second round of voting, agreement was reached.

The Age-Related Eye Disease Study (AREDS)-2 study showed that supplementation with antioxidant nutrients decreases the risk of progression to neovascular AMD [19,58]. The potential benefit of AREDS-based supplementation is also based on several clinical trials with individual supplements [18,22,59,60], and reviewed in [50]. In fact, for many years before AREDS, micronutrition was held to be an integral part of routine management of AMD [17,23]. Moreover, nutritional strategies for secondary prevention of AMD are present in virtually all clinical guidelines [61].

The experts also considered that the best-validated supplementation therapy for patients suffering from AMD with geographic atrophy without central involvement of the fovea is an AREDS-based formulation (statement #13). The last statement on AREDS-based supplementation (#14) held that initiating a supplementation with an AREDS-based formulation in patients at high risk of AMD is more cost effective than no use of supplements and should be advocated. This is largely based on the real-world study by Lee et al. in the UK, wherein initiating AREDS-based supplements in AREDS category 4 patients was found to be both cost-effective and superior to no use of supplements [26].

### 4.5. Moderate and High-Risk Subjects

The remaining statements (#15–25) involved recommendations for monitoring, nutrition, and follow-up in subjects with moderate (STARS^®^ 10–19) and high (STARS^®^ ≥ 20) risk for AMD. Subjects with moderate risk, AREDS category 2, and 55–70 years of age should be asked to carry out self-monitoring (statement 15) and have follow-up every 2–3 years (statement 16), although the recommendation on follow-up frequency was considered of uncertain relevance. The lengthy follow-up time was made in consideration of the low risk of progression at 5 years, although for some panelists it was held that follow-up might be more frequent and that any patient at risk should be asked to carry out self-monitoring. Those with the same risk profile but with age > 70 years should carry out self-monitoring, be recommended specific nutritional supplements, and have annual follow-up (statements 17–19). Some participants felt that even stricter follow-up might be applied in some cases.

The statements for high-risk subjects (STARS^®^ ≥ 20) were divided into those with AREDS category 1 or 2. All subjects with high-risk and with either AREDS category 1 or 2 over the age of 55 should be asked to carry out self-monitoring (statements 20, 21, and 23). High-risk subjects with AREDS category 1 over the age of 70 should be recommended to take specific nutritional supplements (statement 22), as should high-risk subjects with AREDS category 2 over the age of 55 years (statement 24). Independently of age, high-risk subjects with AREDS category 2 should undergo follow-up every 6 months. It was, however, noted that a prospective randomized trial would be needed to confirm the utility of these recommendations in this group of subjects. Lastly, it should be highlighted that these recommendations are broadly in line with those issued by several national societies, such as the American Optometric Association, EURETINA [48], NICE [62], and the American Academy of Ophthalmology [63].

The Delphi methodology is a well-known process that aids in achieving group consensus. An advantage of our methodology is the online procedure adopted. During the two rounds carried out online, response rates were high with copious feedback, in line with a previous report [64].

At the same time, the limitations of the Delphi process warrant comment. First, given the internet-based nature of the present study, the panel members were unable to directly discuss specific wording of the statements. Moreover, the success of the Delphi process is further dependent on the panel selected to participate. While there are no established criteria to select experts and no specific guidance regarding the number of participants, we made a concerted effort to obtain a representative distribution of experts across several countries. The high level of consensus achieved suggests that these limitations are minimal.

## 5. Conclusions

In the present Delphi consensus, statements to support preventive measures in patients at risk of AMD based on STARS^®^ scoring were developed. The recommendations deliver the means to optimize preventive care for patients in different geographical regions. It is hoped that the statements developed will be of benefit to clinicians in daily practice. This is especially relevant given that a proportion of ophthalmologists do not always properly counsel patients on the lifestyle changes to adopt, and, likewise, patients do not always adhere to specific recommendations. Future work should involve translating guidelines into routine clinical practice followed by evaluating their impact on the care of patients.

## Figures and Tables

**Table 1:** Consensus statements on general issues, evaluation tools, general lifestyle advice, and AREDS-based supplementation.

	General Recommendations	IPR	IPRAS	Median	Consensus
1	Intravitreal injections are the first-choice treatment for patients with wet AMD to stop progression of the disease	0	15.0	9.0	Positive agreement
2	Patients at high risk of AMD should receive nutritional supplements to help reduce the risk of progression at an early phase after diagnosis of AMD	2.2	10.9	9.0	Positive agreement
	**Evaluation tools**				
3	Early detection with simple tools is desirable to detect patients at risk of AMD and treat promptly if needed	0	15.0	9.0	Positive agreement
4	The STARS^®^ questionnaire is a valid tool to assess risk of AMD in the general population	1	9.4	8.0	Positive agreement
5	Stratification according to risk of AMD is useful in order to plan lifestyle interventions, give dietary advice and plan follow-up using STARS^®^ and the AREDS category score	1	13.1	8.0	Positive agreement
6	Ophthalmologists should use the STARS^®^ and AREDS classifications in daily practice to evaluate the risk of AMD and to define the best prevention strategy and follow-up for patients	2	11.3	8.0	Positive agreement
	**General lifestyle advice**				
7	All subjects at risk of AMD should be advised to stop smoking, adopt a Mediterranean diet, and carry out regular physical activity	0	15.0	9.0	Positive agreement
8	Increased intake of vegetables, fruit and fish should be actively encouraged in the aging population as <4% of individuals ≥ 55 years of age achieve adequate intake of these food groups	1	13.1	9.0	Positive agreement
9	If patients are unable or unwilling to follow a Mediterranean diet, nutritional supplements should be recommended in subjects at high risk of AMD	2	11.3	9.0	Positive agreement
	**AREDS-based supplementation**				
10	An AREDS-based formulation significantly reduces the risk of developing advanced AMD in the long-term	2	11.3	8.0	Positive agreement
11	An AREDS-based formulation decreases the overall risk of moderate vision loss in the long term	2	11.3	8.0	Positive agreement
12A	An AREDS-based formulation has no significant benefit on the progression of dry AMD or development of geographic atrophy in the long term	4.5	0.9	5.0	No agreement
12B	An AREDS-based formulation may have benefit on the progression of dry AMD or development of geographic atrophy in the long term	3	5.6	7.0	Positive agreement
13	The best-validated supplementation therapy for patients suffering from AMD with geographic atrophy without central involvement of the fovea is an AREDS-based formulation	3.5	4.7	7.0	Positive agreement
14	Initiating supplementation with an AREDS-based formulation in patients at high risk of AMD is more cost effective than no use of supplements and should be advocated	2	11.3	8.0	Positive agreement

**Table 2:** Consensus statements for subjects with moderate and high risk of developing AMD.

	Moderate Risk Subjects (STARS^®^ 10–19)	IPR	IPRAS	Median	Consensus
15	Moderate risk subjects according to STARS^®^ (STARS^®^ score 10–19) and with AREDS category 2 and 55–70 years of age should be asked to carry out self-monitoring (e.g., with Amsler grid)	2.5	10.3	7.5	Positive agreement
16	Moderate risk subjects according to STARS^®^ (STARS^®^ score 10–19) and with AREDS category 2 and 55–70 years should have follow-up every 2 to 3 years	3	3.8	6.5	Uncertain relevance
17	Moderate risk subjects according to STARS^®^ (STARS^®^ score 10–19) and with AREDS category 2 and age > 70 years should be asked to carry out self-monitoring (e.g., with Amsler grid).	2	11.3	8.0	Positive agreement
18	Moderate risk subjects according to STARS^®^ (STARS^®^ score 10–19) and with AREDS category 2 and age > 70 years should be recommended specific nutritional supplements for prevention of AMD	2.5	10.3	8.0	Positive agreement
19	Moderate risk subjects according to STARS^®^ (STARS^®^ score 10–19) with AREDS category 2 and age > 70 years should have annual follow-up	2	11.3	9.0	Positive agreement
	**High risk subjects (STARS^®^ ≥ 20)**				
20	High risk subjects according to STARS^®^ (STARS^®^ ≥ 20), with AREDS category 1 and 55–70 years of age should be asked to carry out self-monitoring (e.g., with Amsler grid)	2.5	10.3	8.0	Positive agreement
21	High risk subjects according to STARS^®^ (STARS^®^ ≥ 20), with AREDS category 1 and age > 70 years should be asked to carry out self-monitoring	3	9.4	8.0	Positive agreement
22	High risk subjects according to STARS^®^ (STARS^®^ ≥ 20), with AREDS category 1 and age > 70 years should be recommended specific nutritional supplements for prevention of AMD	3	9.4	8.0	Positive agreement
23	High risk subjects according to STARS^®^ (STARS^®^ ≥ 20), with AREDS category 2, aged 55 years or more, should be asked to carry out self-monitoring (e.g., with Amsler grid)	1.5	12.2	8.0	Positive agreement
24	High risk subjects according to STARS^®^ (STARS^®^ ≥ 20), with AREDS category 2, aged 55 years or more, should be recommended specific nutritional supplements for prevention of AMD	2	11.3	8.0	Positive agreement
25	High risk subjects according to STARS^®^ (STARS^®^ ≥ 20) with AREDS category 2, independently of age, should have follow up every 6 months	4.5	4.7	7.0	Positive agreement

## Data Availability

Not applicable.
